# Cost‐effectiveness of the Collaborative Care to Preserve Performance in Cancer (COPE) trial tele‐rehabilitation interventions for patients with advanced cancers

**DOI:** 10.1002/cam4.2837

**Published:** 2020-02-23

**Authors:** Colleen F. Longacre, John A. Nyman, Sue L. Visscher, Bijan J. Borah, Andrea L. Cheville

**Affiliations:** ^1^ Division of Health Policy and Management University of Minnesota School of Public Health Minneapolis MN USA; ^2^ Center for the Science of Health Care Delivery Mayo Clinic Rochester MN USA; ^3^ Department of Physical Medicine and Rehabilitation Mayo Clinic Rochester MN USA

**Keywords:** cost effectivness, healthcare utilization, hospitalization, physical function, telecare

## Abstract

**Purpose:**

The purpose of this analysis was to determine the cost‐effectiveness of a Collaborative Care Model (CCM)‐based, centralized telecare approach to delivering rehabilitation services to late‐stage cancer patients experiencing functional limitations.

**Methods:**

Data for this analysis came from the Collaborative Care to Preserve Performance in Cancer (COPE) trial, a randomized control trial of 516 patients assigned to: (a) a control group (arm A), (b) tele‐rehabilitation (arm B), and (c) tele‐rehabilitation plus pharmacological pain management (arm C). Patient quality of life was measured using the EQ‐5D‐3L at baseline, 3‐month, and 6‐month follow‐up. Direct intervention costs were measured from the experience of the trial. Participants’ hospitalization data were obtained from their medical records, and costs associated with these encounters were estimated from unit cost data and hospital‐associated utilization information found in the literature. A secondary analysis of total utilization costs was conducted for the subset of COPE trial patients for whom comprehensive cost capture was possible.

**Results:**

In the intervention‐only model, tele‐rehabilitation (arm B) was found to be the dominant strategy, with an incremental cost‐effectiveness ratio (ICER) of $15 494/QALY. At the $100 000 willingness‐to‐pay threshold, this tele‐rehabilitation was the cost‐effective strategy in 95.4% of simulations. It was found to be cost saving compared to enhanced usual care once the downstream hospitalization costs were taken into account. In the total cost analysis, total inpatient hospitalization costs were significantly lower in both tele‐rehabilitation (arm B) and tele‐rehabilitation plus pain management (arm C) compared to control (arm A), (*P* = .048).

**Conclusion:**

The delivery of a CCM‐based, centralized tele‐rehabilitation intervention to patients with advanced stage cancer is highly cost‐effective. Clinicians and care teams working with this vulnerable population should consider incorporating such interventions into their patient care plans.

## Graphical abstract

Cost‐effectiveness analyses conducted in parallel with a randomized clinical trial of 516 patients with late‐stage cancer assigned to: (a) a control group, (b) tele‐rehabilitation, and (c) tele‐rehabilitation plus pharmacological pain management found the second arm to be dominant, with an incremental cost‐effectiveness ratio of $15 494/QALY. When reduced health‐care utilization was considered, the tele‐rehabilitation arms were cost saving.

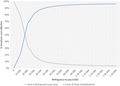

## 1 INTRODUCTION

"Health‐care costs related to cancer increase at a rate that far exceeds inflation, leaving few patients unaffected.^1^, ^2^, ^3^ The consequences of this escalation include an almost threefold higher rate of personal bankruptcy among patients with cancer,^4^ with reports establishing “financial toxicity,” that is, problems related to the cost of treatment,^5^ as a clear threat to patients’ survival.^4^, ^6^ Spiraling drug costs have been highlighted as an important contributor.^7^, ^8^ However, expenses rapidly mount from diverse sources including diagnostic testing, clinician visits, and symptom management,^9^ suggesting that an exclusive focus on any single charge category will fail to meaningfully improve the situation. Payment‐based solutions, such as bundled care and episode‐based reimbursement, have been slow to gain traction,^10^, ^11^ and credible targets to ease patients’ financial strain remain elusive.

Given the mounting concern over cancer costs, surprisingly, limited attention has been directed to potentially remediable drivers of patients’ requirement for health‐care services, namely pain and disablement. Associations between patients’ pain and their hospital and emergency department usage have been long recognized,^12^, ^13^ particularly among cancer cohorts.^14^ However, linkages between patients’ functional status and their health‐care consumption, though robust, have received less attention. Further, until recently, associations of health‐care costs with adverse symptoms and loss of function were considered neither causal nor remediable. These assumptions have been challenged by reports of reduced hospital and emergency department service utilization among patients randomized to quality of life‐ and symptom‐directed interventions that improve pain and/or function.^15^, ^16^, ^17^ The recently reported Collaborative Care to Preserve Performance in Cancer (COPE) was notable among these efforts as the first to significantly reduce hospital lengths of stay and requirements for post‐acute care by targeting pain and function.^18^ Moreover, the COPE trial enrolled patients with advanced stage cancers, a population whose hospitalizations account for up to two‐thirds of their health‐care costs.

Care delivery to alleviate pain and improve function can be resource intensive. Whether these interventions have the potential to be cost neutral or saving is under‐researched. The few reported cost‐effectiveness analyses of interventions targeting cancer pain are promising as they suggest incremental cost‐effectiveness ratios (ICERs) within conventional willingness‐to‐pay thresholds.^19^, ^20^, ^21^ As yet, we lack similar estimates for interventions that target function along or in association with pain. This manuscript reports ICERs and cost‐effectiveness analysis for the COPE trial's two intervention arms, and a secondary cost analysis among participants represented in the Rochester Epidemiology Department for whom comprehensive cost capture was possible.

## 2 MATERIALS AND METHODS

### 2.1 Overview

The randomized clinical COPE trial assigned 516 patients who were experiencing functional limitations to: (a) a control group (arm A), (b) tele‐rehabilitation (arm B), and (c) tele‐rehabilitation plus pharmacological pain management (arm C). Clinical outcomes of this trial have been reported elsewhere.^18^ All arms underwent automated home‐based monitoring of physical functioning and pain via telephone and/or internet, with reporting of these data to their care teams. Participants in arms B and C received centralized tele‐rehabilitation based on the collaborative care model (CCM) provided by a physical therapist (PT) physician team. The tele‐rehabilitation intervention included a pedometer‐based walking program and a resistive exercise program, both validated.^16^, ^22^ Direction was provided remotely via telephone during one‐to‐one calls between participants and the study PTs. Arm C participants also received similarly delivered, nurse‐coordinated pain management based on an iteration of the CCM previously validated among patients with cancer.^17^ Participants who did not complete their scheduled assessments, did not adhere to the recommended frequency of exercise sessions, or reported functional losses were contacted by a PT for further follow‐up.

The COPE trial was conducted at three academic medical centers located in the upper Midwest, Southwest, and Southeast sections of the United States. All sites were part of a single health‐care system and National Cancer Institute‐designated comprehensive cancer center. COPE trial participants were seen for at least two visits in an outpatient medical oncology or hematology clinic at one of the sites.

Patient quality of life was measured using the EQ‐5D‐3L at baseline, and at 3‐ and 6‐month follow‐up. The EQ‐5D‐3L has robust support for validity in populations with advanced malignancies.^23^, ^24^ Direct intervention costs were measured from the experience of the trial. Participants’ hospitalization data were obtained through abstracting the medical record, and costs associated with these encounters were estimated from unit cost data and hospital‐associated utilization information found in the literature. A secondary analysis of total utilization costs was conducted for the subset of COPE trial patients living in Olmsted County, whose data were available from the Rochester Epidemiology Project (REP).

### 2.2 The decision‐analysis model

**Figure 1:**
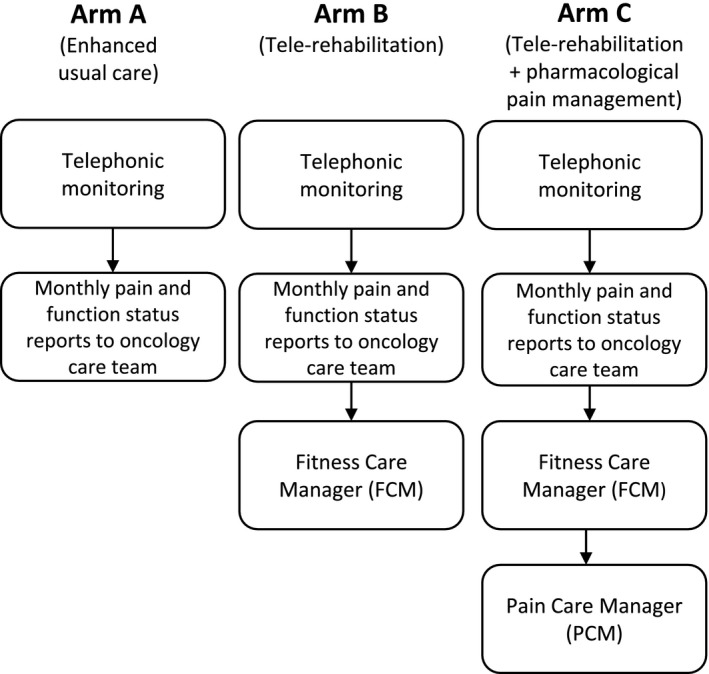
Overview of the three Collaborative Care to Preserve Performance in Cancer Trial arms

A decision‐analytic model was constructed using TreeAgePro software (2017) to assess the three intervention strategies examined in the COPE trial (Figure 1). Participants were assumed to receive the full intervention of the arm to which they were assigned. Participants could experience health‐care utilization in the form of hospitalizations. Each hospitalization was associated with a length of stay (in days).

### 2.3 Parameter estimates

Table 1 provides the full list of parameter estimates and distributions used in the model.

**Table 1:** Parameter estimates for the decision model

Parameter	Arm	Mean (base value)	Standard deviation	Distribution	Source
Effectiveness					
EQ‐5D‐3L index change from A	A	—	**—**	**—**	Cheville et al^18^
	B	0.04	0.02	Normal	
	C	0.03	0.02	Normal	
Costs					
Intervention costs					Cheville et al^18^
Instruction DVDs	B+C	$18.00			
Elastic resistance bands	B+C	$11.55			
Pedometers	B+C	$24.64			
FCM call time	B	$100.76	$49.13	Gamma	
FCM and PCM call time	C	$128.32	$82.94	Gamma	
PCM meeting	C	$87.68			
Utilization					
Probability of hospitalization	A	0.192			Cheville et al^18^
	B	0.221			
	C	0.233			
Hospital length of stay (days)	A	7.4	9.3	Gamma	Cheville et al^18^
	B	3.5	4.3	Gamma	
	C	5	7.2	Gamma	
Utilization Costs					
Cost of inpatient hospital day	A, B, C	$2409.00			Kaiser Family Foundation^25^

#### 2.3.1 Effectiveness

The incremental utility gain per arm was measured by the incremental change in EQ‐5D‐3L score per arm over the course of the trial. We assumed a linear increase in effectiveness between baseline and 6 months and that utility gains only lasted until the conclusion of the trial. The incremental utility gain over the 6‐month trial period was then converted into quality‐adjusted life years (QALYs) for the cost‐effectiveness analysis.

#### 2.3.2 Costs

##### Intervention costs

Fixed intervention costs for participants in trial arm B included: (a) the cost of instructional DVDs and elastic resistance bands used by the “Rapid Easy Strength Training” resistive exercise program; and (b) the cost of pedometers associated with the “First Step Program”, an incremental pedometer‐based walking program. Variable intervention costs for participants in trial arm B included time spent on the phone with a PT, the “fitness care manager” (FCM), who has specialized in cancer rehabilitation. The dates and lengths of these telephone calls were recorded, and the cost of the calls were calculated based on the salaries of the FCMs participating in the trial. In addition to the fixed and variable costs associated with arm B, the variable costs for arm C included the time spent on the phone with a nurse “pain care manager” (PCM), who reached out to those patients with high pain scores and provided treatment recommendations to the study participant's oncologic, hematologic, or primary care team, who were responsible for prescribing all medications. The dates and lengths of telephone calls with patients were recorded, and these time costs were calculated, based on the salaries of the PCMs participating in the trial. Consultative meetings were held biweekly and the time spent in these meetings was not tracked by the patient; therefore, PCM time costs for meetings are considered fixed.

##### Utilization costs

We estimated health‐care utilization costs in two ways: (a) by applying unit cost estimates from the literature to collected hospital utilization data and (b) collecting cost data from a subset of the patient population of the COPE trial tracked by the REP.

##### Estimated hospitalization costs from the literature

The COPE trial tracked utilization (participant hospitalizations and hospital lengths of stay), but did not track the total costs associated with these visits. As a result, hospitalization costs were estimated using unit costs from the literature. Because patients in the COPE trial with stage III‐IV cancers are likely to be significantly different from the overall population of hospital patients surveyed in most cost studies, estimates of cancer treatment costs were not included. Instead, because we had participant length of stay, we used a measure of the hospital‐adjusted expenses per inpatient day, estimated from the Kaiser Family Foundation in 2015.^25^ This figure provides an estimate of expenses incurred by the hospital to provide a day of inpatient care, but does not include the cost of specific services (procedures or treatments) provided to the patient.

##### Estimated costs from the REP

While complete utilization cost data were not available for all trial participants, these data were available from the REP for a subset of COPE trial participants who were living in Olmsted County, Minnesota. The REP is a medical records‐linkage system that tracks virtually all health‐care services provided to the residents of Olmsted County, Minnesota by Olmsted County‐based providers.^26^, ^27^ The population counts obtained by the REP Census match those obtained by the US Census, indicating that the population of the county is captured by the system.^28^ The REP tracks the vital and residential status of all Olmsted residents, and capture billing codes for all their health care.^28^ From the REP data, we estimated the total costs, cancer‐related inpatient costs, cancer‐related outpatient costs, cancer‐related emergency department costs, and all‐cause clinic costs. The REP reports *standardized costs,* which are created from an internally developed algorithm that applies Medicare reimbursement to professional services, multiplies the charges for hospital services by the appropriate Medicare cost report cost‐to‐charge ratios, and adjusts for inflation with the GDP implicit price deflator.^29^ This standardization process, however, renders these costs incomparable with the costs of the direct intervention because of the proprietary nature of the standardization algorithm. Therefore, we did not calculate cost‐effectiveness measures from these data. Instead, we calculated Wilcoxon scores (rank sums) for these cost measures and determined whether these costs varied significantly across the three arms using the Kruskal‐Wallis nonparametric analysis of variance test.^30^

#### 2.3.3 Cost‐effectiveness measures

##### Incremental cost‐effectiveness ratio

We used the ICER^31^ as a point estimate of the cost‐effective treatment strategy.

##### Probabilistic sensitivity analysis

Where appropriate, we then ran a probabilistic sensitivity analysis (PSA) of 100 000 simulations using the means, assumed distribution and confidence intervals from the cost and effectiveness results to determine and construct the cost‐effectiveness acceptability curve (CEAC) associated with the treatment strategies. The CEAC allows us to summarize which strategy is more likely to be cost‐effective at various willingness‐to‐pay thresholds.^32^

## 3 RESULTS

Table 2 compares the demographic and clinical characteristics of the patients in the COPE trial who were and were not captured in the REP data. Participants in the REP data were more likely than non‐REP patients to receive chemotherapy as part of their cancer treatment; otherwise, the two cohorts did not differ significantly in their sociodemographic characteristics, clinical presentation, and outcomes reported at baseline.

**Table 2:** Collaborative Care to Preserve Performance in Cancer trial participant baseline characteristics

Patient characteristics	All (n = 516)	REP (n = 104)	Non‐REP (n = 412)	*P* value*
Trial arm, no. (%)				.48
A (Enhanced usual care)	172 (33.3)	30 (28.9)	142 (34.5)	
B (Tele‐rehabilitation)	172 (33.3)	35 (33.7)	137 (33.3)	
C (Tele‐rehabilitation + pain management)	172 (33.3)	39 (37.5)	133 (32.3)	
Sociodemographic				
Age, mean (SD)	65.6 (11.1)	65.3 (11.5)	65.7 (11.0)	.78†
Female sex, no. (%)	257 (49.8)	55 (52.9)	202 (49.0)	.48
Race, no. (%)				.32‡
White	492 (95.3)	102 (98.1)	390 (94.7)	
Non‐white	24 (4.8)	2 (1.9)	22 (5.3)	
Ethnicity				.44‡
Hispanic or latino	28 (5.4)	3 (2.9)	25 (6.1)	
Marital status, no. (%)				.25
Married or partnered	410 (79.5)	83 (79.8)	327 (79.4)	
Widowed	33 (6.4)	8 (7.7)	25 (6.1)	
Divorced or separated	36 (7.0)	6 (5.8)	30 (7.3)	
Single	37 (7.2)	7 (6.7)	30 (7.3)	
In‐home caregiver without disability, no. (%)	511 (99.0)	101 (97.1)	410 (99.5)	.06‡
Community‐based oncological care team, no. (%)	289 (56.1)	53 (51.0)	236 (57.4)	.24
Clinical, non‐cancer				
Functionally relevant comorbidities, no. (%)				
Coronary artery disease	64 (12.4)	15 (14.4)	49 (11.9)	.48
Neuropathy	112 (21.7)	17 (16.4)	95 (23.1)	.14
Cancer				
Bone metastases, no. (%)	264 (51.3)	46 (44.2)	218 (53.0)	.11
Treatment at baseline, no. (%)				
Chemotherapy	242 (46.9)	58 (55.8)	184 (44.7)	.04
Biological	161 (31.2)	25 (24.0)	136 (33.0)	.08
Hormone	216 (41.9)	37 (35.6)	179 (43.4)	.15
Baseline outcomes				
Patient‐reported outcomes, mean (SD)				
AM‐PAC‐CAT basic mobility	60.3 (3.6)	60.6 (3.3)	60.3 (3.7)	.34†
EQ‐5D‐SL	0.8 (0.1)	0.8 (0.1)	0.8 (0.1)	.13†
BPI total interference	2.2 (2.1)	2.1 (2.1)	2.2 (2.1)	.55†

* *P* values calculated using the Chi‐squared test, unless otherwise noted.

^†^ *P* value calculated using linear regression.

^‡^ *P* value calculated using the Fisher's exact test for cell size <5.

The mean cost of the intervention per patient was $154.94 in arm B and $270.18 in arm C (Table 3). Staff time spent on calls with patients was the largest contributor to the intervention costs (65% in arm B and 48% in arm C), followed by the time spent in consultative meetings by the PCMs in arm C (32% of arm C costs). The mean QALY gain in arm B was 0.01 (3.65 days of the equivalent of perfect health) and in arm C was 0.0075 (2.74 such days), both compared to control arm A.

**Table 3:** Incremental cost‐effectiveness ratio (ICER) point estimates for the intervention‐only and intervention plus hospitalization models

Arm	Description	Mean cost	Mean effectiveness (QALY) gain	ICER
*Intervention‐only*				
A	Enhanced usual care	$0.00	0	—
C	Tele‐rehabilitation plus pain management	$270.18	0.0075	dominated
B	Tele‐rehabilitation	$154.94	0.01	15 494
*Intervention plus hospitalization*				
A	Enhanced usual care	$3423.13	0	dominated
C	Tele‐rehabilitation plus pain management	$3077.54	0.0075	dominated
B	Tele‐rehabilitation	$2018.54	0.01	cost savings

We present two types of results for each of our two cost‐effectiveness analyses: (a) the expected (mean) costs and effectiveness (QALYs) and the ICER derived from these; and (b) the results of the PSA. We then provide results of the cost analysis of the REP data.

### 3.1 Intervention‐only

**Figure 2:**
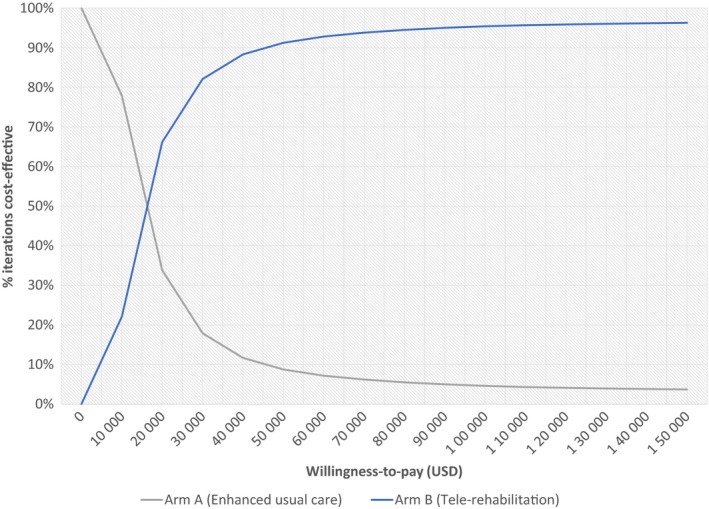
Cost‐effectiveness acceptability curve, intervention‐only analysis (Arm A vs Arm B). This figure shows the percentage of iterations in the probabilistic sensitivity analysis that each intervention (enhanced usual care and tele‐rehabilitation) was shown to be cost‐effective at various willingness‐to‐pay thresholds between $0 and $150 000

Our initial model looked only at the direct costs of the intervention. The mean incremental cost of tele‐rehabilitation (arm B) over enhanced usual care (arm A) was $154.94 per patient, while the corresponding mean effectiveness gain was 0.01 QALYs, for an ICER of $15 494/QALY (Table 3). A strategy is considered dominated if it is both more costly and less effective than another strategy. Arm C was found to be more costly and less effective than arm B; therefore, tele‐rehabilitation plus pain management (arm C) was dominated by tele‐rehabilitation alone (arm B). At the $100 000 willingness‐to‐pay threshold, tele‐rehabilitation (arm B) was found to be the cost‐effective strategy in 95.4% of simulations, compared to enhanced usual care (arm A), which was the cost‐effective strategy in only 4.6% of simulations. Because of the relatively low overall cost of the intervention, tele‐rehabilitation became cost‐effective at willingness‐to‐pay thresholds as low as $15 494, as shown in the cost‐effectiveness acceptability curve (Figure 2).

### 3.2 Intervention plus hospitalization costs

Our second model incorporated the probabilities of hospitalizations and estimated lengths of stay, and associated costs estimated from the literature. The mean cost for tele‐rehabilitation (arm B) was $1420 lower than for enhanced usual care (arm A) while the mean effectiveness gain remained at 0.01 QALYs (Table 3). Thus, the tele‐rehabilitation intervention was found to be cost saving compared to enhanced usual care once the downstream hospitalization costs were taken into account. Tele‐rehabilitation plus pain management (arm C) was again dominated by tele‐rehabilitation (arm B), as arm C was again more costly and less effective.

### 3.3 Full cost data for REP patients

Table 4 presents the results of the cost analysis conducted on the subset of COPE trial participants represented in the REP data. Among these participants, patients in the tele‐rehabilitation arm (arm B) had mean total costs that were 13.6% lower than patients in the tele‐rehabilitation plus pain management arm (arm C) and 25% lower than patients in the enhanced usual care arm (arm A), though neither of these results were statistically significant. However, total inpatient hospitalization costs differed significantly across the three trial arms (*P* = .048). Outpatient, emergency department, and clinic costs did not differ significantly across the three arms. Importantly, the REP‐based ranking of costs matched our previous ranking of costs based on hospital length of stay.

**Table 4:** Cost analysis of subset of Collaborative Care to Preserve Performance in Cancer trial participants represented in the Rochester Epidemiology Project data

	Mean (SD) costs per arm (USD)			
	Arm A	Arm B	Arm C	*P*‐Value*
Total	18 838 (16 620)	14 130 (17 918)	16 351 (20 749)	.079
Inpatient hospitalization	7841 (16 715)	1189 (3656)	2463 (10 100)	.048
Outpatient	4212 (6569)	3782 (6116)	6473 (12 806)	.637
Emergency department	324 (994)	82 (311)	125 (592)	.638
Clinic	6461 (6615)	9076 (14 807)	7290 (10 386)	.284

* *P*‐values calculated using the Kruskal‐Wallis test.

## 4 DISCUSSION

Care delivered to individuals with advanced stage cancers represents a large cost burden to the health‐care system, in part due to patients’ adverse symptoms and loss of function. Tele‐rehabilitation, based on the CCM model, is a low‐cost, low‐intensity intervention that was found in the COPE trial to improve patients’ quality of life, function, and pain. We conducted cost‐effectiveness analyses comparing the COPE trial arms and found that the tele‐rehabilitation intervention was cost‐effective at a willingness‐to‐pay threshold of only $15 494/QALY (Figure 2), which is considerably below standard willingness‐to‐pay thresholds of $50 000 or $100 000 established in the medical and health economics literatures.^21^ Additionally, the tele‐rehabilitation intervention became cost savings when downstream hospitalization costs were taken into account.

The COPE trial intervention is a patient‐centric approach that meets requirements, identified by the National Academy of Medicine, to improve the experiences and outcomes of patients with cancer. As payment for cancer care evolves, oncology care providers will likely be incentivized to address patients’ functional losses through bundled reimbursement initiatives, not only to avoid costly downstream disablement, but also because functional autonomy is an unwavering patient priority. Insurance coverage of the telecare approach employed in COPE is, at present, limited by constraints on federal and commercial payers. However, the accelerating shift away from fee‐for‐service toward value‐based reimbursement likely will allow for more flexible delivery approaches that do not confine payment to clinic‐based care.

Results from the COPE trial^18^ suggest that the cost savings may have stemmed from the fact that, while patients in the tele‐rehabilitation arms were more likely to have a hospitalization than patients receiving enhanced usual care, these hospitalizations were more likely to have been planned in advance for treatment and not to require intensive care unit (ICU) admissions, thereby resulting in significantly lower lengths of stay.

Because our model used a conservative estimate of hospitalization costs, we have reason to suspect that our estimate may represent a lower bound of cost savings generated by this intervention. For example, our hospitalization model did not specifically incorporate ICU costs due to limitations of the ICU data; however, we can assume the higher ICU admissions experienced by the enhanced usual care arm would have contributed significantly to their overall costs. Patients in the tele‐rehabilitation arm (arm B) were also more likely to be discharged home rather than to a skilled nursing facility, which we can also assume would result in additional cost savings. Our secondary analysis of the subset of patients included in the REP data suggests further cost savings from the tele‐rehabilitation intervention when total health‐care utilization costs are taken into consideration, although we acknowledge that these data are limited given the small sample size of patients included in the REP subset and the statistically nonsignificant results for cost categories other than inpatient hospitalizations. However, a strength of the REP data is its comprehensive charge capture across all payers. Additional research that is able to incorporate these data for an entire study sample would be useful in improving model precision, as well as the generalizability of the results.

Another limitation of our study is that the time horizon of the decision model was limited to the intervention period (6 months) for each participant. As a result, we were not able to capture any longer term impact on patient quality of life, health‐care utilization, or costs. However, this limitation is mitigated somewhat given the life expectancy of individuals in this patient population and the fact that the intervention aimed to improve the quality of life, rather than to extend survival.

## 5 CONCLUSION

The delivery of a CCM‐based, centralized tele‐rehabilitation intervention to patients with advanced stage cancer and functional limitations appears to be highly cost‐effective. Clinicians and care teams working with this vulnerable population should consider incorporating such interventions into their patient care plans.
